# Surface chromium on Terracotta Army bronze weapons is neither an ancient anti-rust treatment nor the reason for their good preservation

**DOI:** 10.1038/s41598-019-40613-7

**Published:** 2019-04-04

**Authors:** Marcos Martinón-Torres, Xiuzhen Li, Yin Xia, Agnese Benzonelli, Andrew Bevan, Shengtao Ma, Jianhua Huang, Liang Wang, Desheng Lan, Jiangwei Liu, Siran Liu, Zhen Zhao, Kun Zhao, Thilo Rehren

**Affiliations:** 1 Department of Archaeology, University of Cambridge, Cambridge, United Kingdom; 2 UCL Institute of Archaeology, London, United Kingdom; 3 Emperor Qin Shihuang’s Mausoleum Site Museum, Xi’an, P.R. China; 4 Institute of Historical Metallurgy and Materials, University of Science and Technology Beijing, Beijing, P. R. China; 5 Science and Technology in Archaeology and Culture Research Center, The Cyprus Institute, Nicosia, Cyprus

## Abstract

For forty years, there has been a widely held belief that over 2,000 years ago the Chinese Qin developed an advanced chromate conversion coating technology (CCC) to prevent metal corrosion. This belief was based on the detection of chromium traces on the surface of bronze weapons buried with the Chinese Terracotta Army, and the same weapons’ very good preservation. We analysed weapons, lacquer and soils from the site, and conducted experimental replications of CCC and accelerated ageing. Our results show that surface chromium presence is correlated with artefact typology and uncorrelated with bronze preservation. Furthermore we show that the lacquer used to cover warriors and certain parts of weapons is rich in chromium, and we demonstrate that chromium on the metals is contamination from nearby lacquer after burial. The chromium anti-rust treatment theory should therefore be abandoned. The good metal preservation probably results from the moderately alkaline pH and very small particle size of the burial soil, in addition to bronze composition.

## Introduction

**Figure 1:**
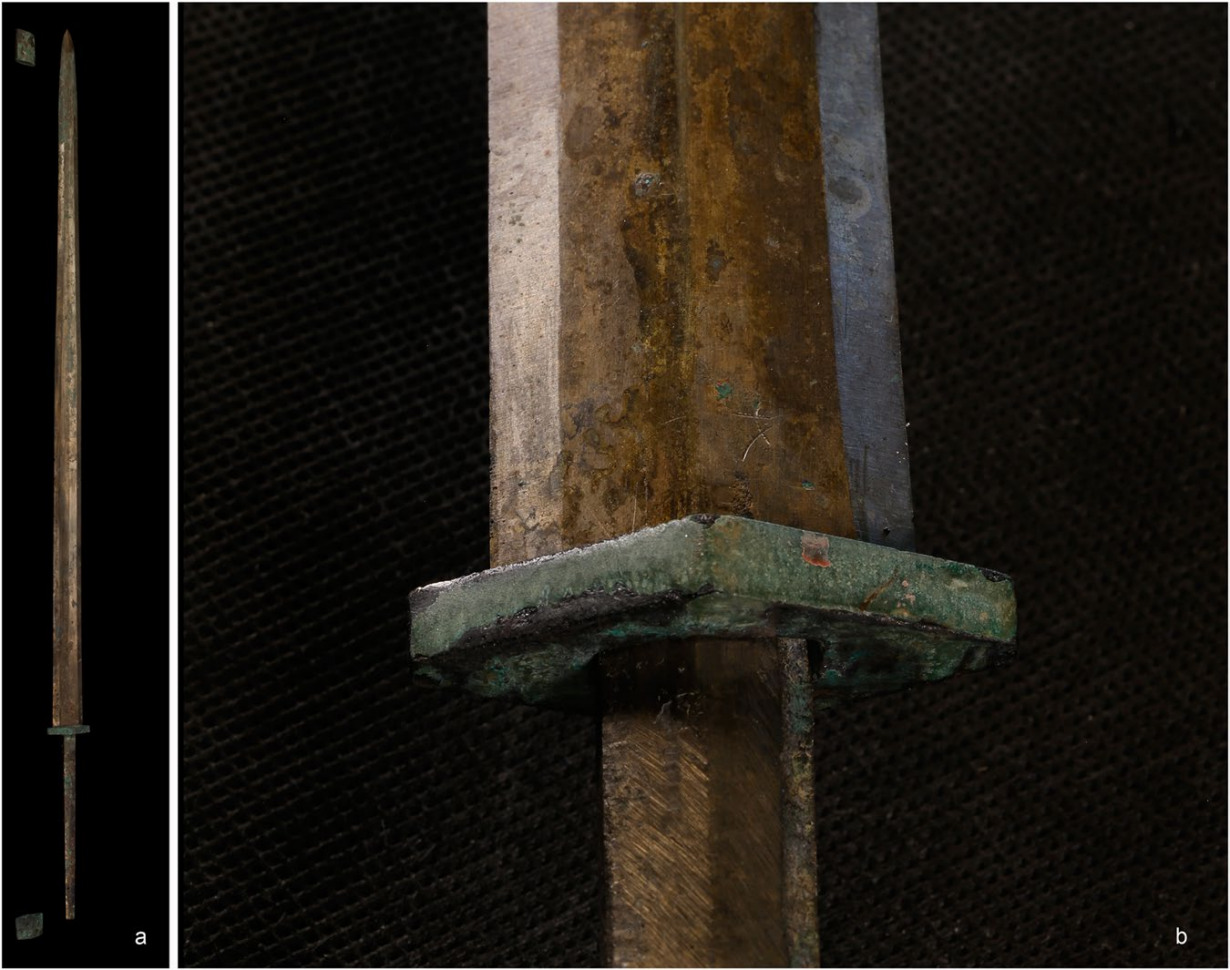
(**A**) Bronze sword from the Terracotta Army with associated fittings from the grip and the scabbard. (**B**) Detail of the grip and blade from another sword.

The Qin Terracotta Army of Xi’an is an array of life-sized, realistic ceramic figures representing warriors, stationed in three large pits within the mausoleum of Qin Shihuang (259–210 BC), the first emperor of a unified China. Over two thousand ceramic warriors have been excavated so far, and it is estimated that several thousand more remain buried^1–4^. These warriors were armed with fully functional weapons made primarily of bronze. From the partial excavation of Pit 1 alone, dozens of spears, lances, swords and hooks have been recovered, in addition to over 80 ferrules that would have been attached to the distal end of shafted weapons, over 260 crossbow triggers and as many as 40,000 arrowheads, typically found in bundles of one hundred that originally were the contents of one warrior’s quiver. In most cases, the organic components of the weapons, such as wooden shafts, quivers, scabbards or crossbow stocks, have largely decayed. However, the preservation of the bronze is remarkably good overall, with many of the weapons displaying shiny, almost pristine surfaces and sharp blades (Fig. 1).

Since the first excavation of these bronze weapons in the 1970s, researchers highlighted the arguably exceptional state of preservation of the weapons after over two thousand years buried^1:306–7&341–8,^^5^. They hypothesised that Qin weapon-makers could have utilised some kind of anti-rust technology to prevent them from decaying in their afterlife. The identification of chromium oxide enrichment on the surface of a few weapon samples led to suggestions that an early precedent of so-called chromate conversion coating (CCC)^6,7^ could have been applied by Qin craftspeople in order either to prevent corrosion and/or to darken the weapon surface colours. Furthermore, laboratory experiments demonstrated that CCC was viable given the raw materials and technology available at the time, even though replication experiments were not conclusive^5^. After that, references to ‘corrosion resistance’ aided by chromium and the ‘highly developed technology’ of weapon-makers appeared in excavation reports and other publications, which have been further amplified by translation into English and other languages, and reproduction in popular media^1,2^. Two studies have challenged this hypothesis in academic publication: based on metallographic observations of a small number of samples, researchers suggested that the better preservation of some weapons could be the result of a compact tin-rich surface layer that acted as a barrier against bronze corrosion; these authors further proposed that the surface chromium derived from contamination from the soil fill of the pits^8,9^. However, aside from the limitations of a very small sample size, these researchers failed to demonstrate the presence of chromium in the soil itself, or to explain the mechanism whereby this element might have migrated onto the weapon surfaces. Thus the proposition that CCC was deliberately carried out by Qin weapon makers has remained a perplexing issue.

Our research set out to revisit this matter by addressing the following questions: What is the frequency of chromium presence on the surface of the weapons? Is there a correlation between surface chromium presence and bronze preservation? What is the source of chromium? Is surface chromium the result of deliberate action? Here we present analyses of a large sample of archaeological weapons, soils and associated materials, experimental replications of chromium surface treatments, and accelerated ageing experiments of bronzes. Our results demonstrate that the chromium enrichment on the surface of some weapon parts is not the consequence of a deliberate treatment but the result of post-depositional contamination, and that the source of chromium is the lacquer abundantly documented at the site. Further, we propose for the first time that, rather than chromium presence, one key reason for the good preservation of the bronzes may be in the quality of the local soil.

## The Bronze Weapons and Their Surfaces

**Figure 2:**
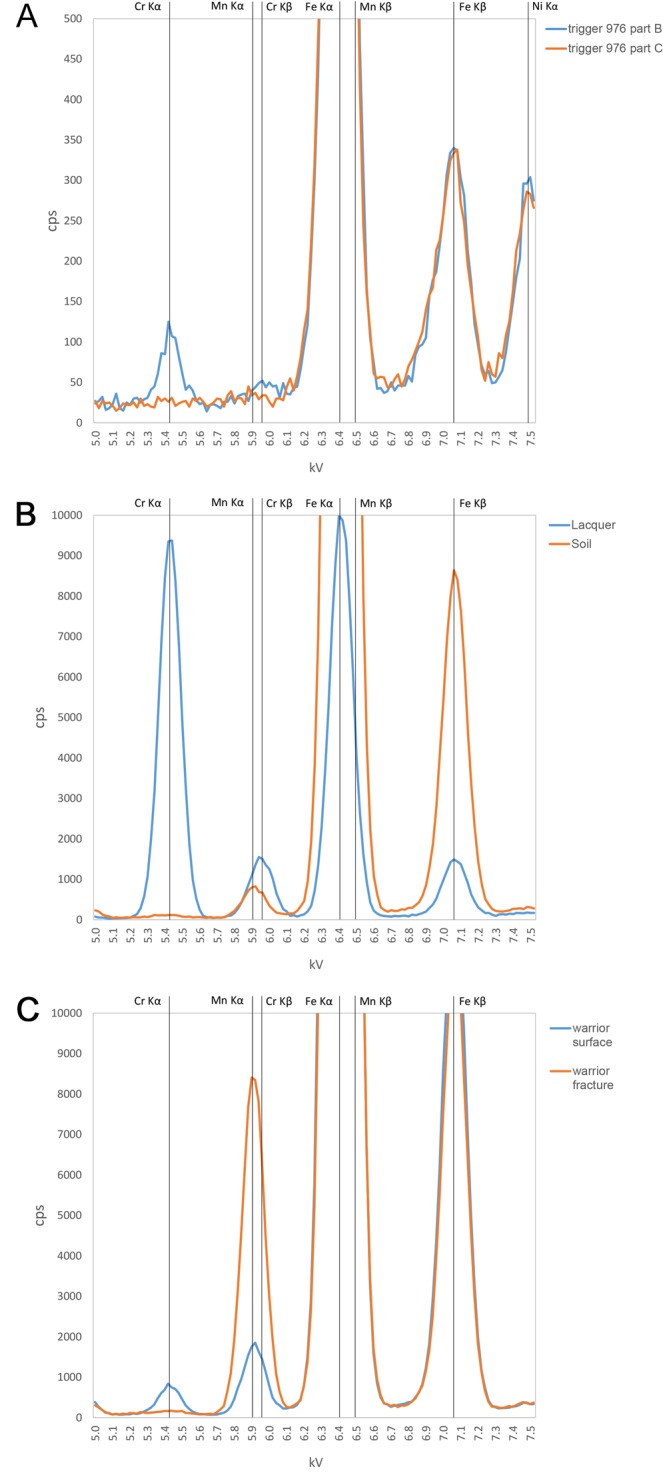
Examples of pXRF spectra comparisons illustrating the variable results for chromium. (**A**) Two parts of the same bronze trigger. (**B**) Lacquer vs soil sample. (**C**) Outer surface vs fracture surface on a ceramic warrior (see Supplementary Material for more results).

Except for a very small number of weapon parts made of iron, all the metal components of the Terracotta Army weapons are made of bronze, i.e. an alloy of copper and tin (~5–25wt% Sn). Antimony, arsenic, and lead are often present but in relatively low concentrations (<3 wt% each), with iron, sulphur and nickel as the additional minor elements most commonly detected in their bulk compositions^10^. Surface portable XRF analyses of 464 weapon parts identified chromium ≥0.1% in 37 (8%) of them, confirming that the presence of this element on the metal surfaces is not universal. Our large sample size allowed us, for the first time, to observe patterns in the presence/absence of chromium: this element was frequently detected in pommels and other fittings of swords and lances (88% of the samples in this category), the handles and tumblers of crossbow triggers (75%), and the ferrules placed at the distal end of long weapon shafts (67%). Conversely, it was only rarely detected in trigger levers or bolts (13–17%), arrow heads (1.5%) and arrow tangs (0.5%), and in none of the blades from swords and lances (Tables 1 and S2; Fig. 2A).

**Table 1:** Frequency of chromium detection by pXRF on the surface of the bronze weapon parts analysed.

	Analysed	Surface Cr	%
Arrow heads	194	3	2
Arrow tangs	196	1	1
Ferrules	6	4	67
Sword/lance blade	8	0	0
Sword/lance fittings	8	7	88
Trigger part A	12	9	75
Trigger part B	12	9	75
Trigger part C	12	2	17
Trigger part D	8	1	13
Trigger part E	8	1	13
Total	464	37	8

**Figure 3:**
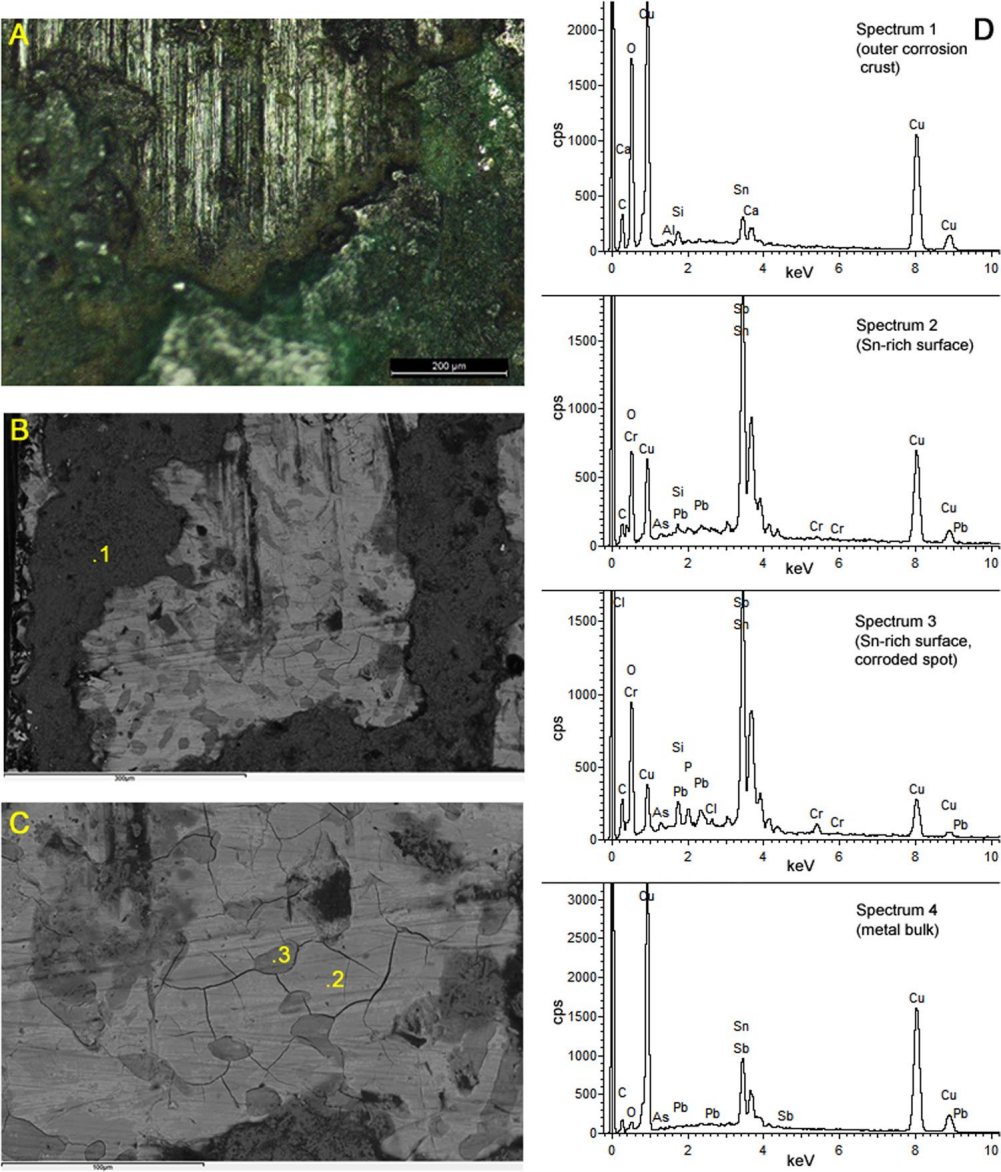
Characterisation of the surface of a bronze sword. (**A**) Metal surface with parallel striations from filing, partly overlaid by a green patina. (**B**,**C**) SEM-BSE detail of the surface, illustrating the points for EDS analysis. (**D**) Example EDS spectra for the various phases analysed on the surface of the sword (spectra 1–3) compared to the fresh metal in section (spectrum 4). Note the presence of chromium exclusively in the Sn-enriched surface.

**Figure 4:**
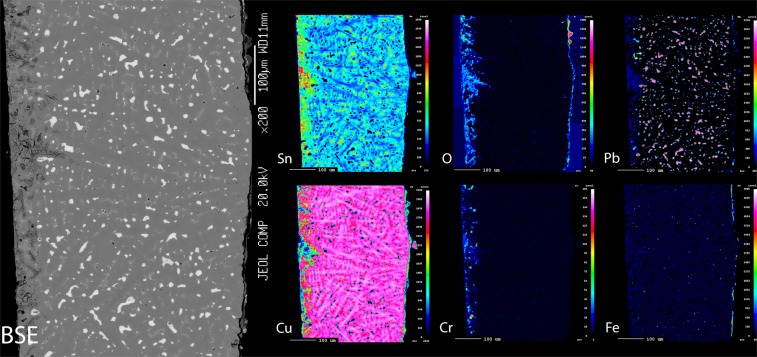
Backscattered electron image and X-ray maps from a cross-section of a scabbard fitting, with the inner surface to the left and outer surface to the right. Note higher concentration of chromium towards the inner surface, which is more oxidised and also enriched in tin and lead.

The weapon surfaces often show toolmarks from the filing away of excess metal after casting, and bladed items additionally display parallel striations resulting from fine polishing and sharpening^11^. Metallographic sections generally show copper-rich alpha dendrites in a tin-rich alpha + delta eutectoid matrix, as typical of cast bronzes that have undergone little or no mechanical work after casting^10^. The only signs of deformation are strain lines towards the surfaces of objects that have undergone filing and polishing. Small lead globules and copper-iron sulphide inclusions can also be observed. Some bronzes have a thin (2–15 µm), relatively compact, dark surface layer that is dominated by tin oxide, sometimes with lead oxide as an accessory compound. This is especially prevalent in objects with a higher bulk tin content (>10% Sn), where loss of surface copper is known to facilitate the development of a stable patina^12–14^. If present, the chromium enrichment is particularly noticeable in this layer (up to 2.5 wt% Cr detected by SEM-EDS), invariably associated to oxides rather than the metal phase. SEM-EDS spot analyses show that the concentration of chromium varies across this layer, with slightly higher concentrations in more corroded spots - but no discrete chromium-rich crystals were identified via microscopy or Raman analyses. On top of this layer, patches of a porous and sometimes thicker layer can be found, typically dominated by green copper carbonates with soil particles embedded, and variously enriched in silica, lime or iron oxide from the soil (Fig. 3). Of particular interest is sample 3801 (TS20), from the metal tip of the scabbard of a sword: EPMA X-ray mapping clearly shows that the outer surface is chromium-free and significantly less corroded, although it bears a thin layer of iron-rich contamination from the soil; conversely, the inner surface shows more substantial corrosion, and the association of the oxides of tin, lead and chromium to corroded features can be clearly noticed (Fig. 4).

## Post-Depositional Chromium on the Weapons

**Figure 5:**
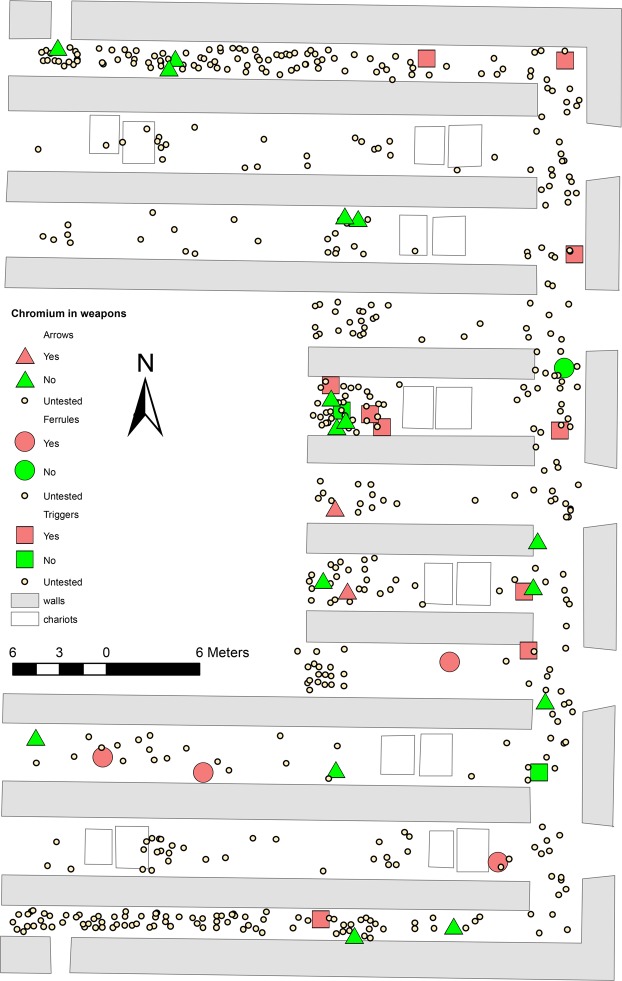
Spatial distribution of Cr-rich weapons. Distribution map of the triggers, ferrules and arrow bundles excavated in the easternmost trenches of Pit 1, noting those analysed by pXRF and highlighting the samples found to contain chromium.

Our previous research has shown that the weapons in Pit 1 occur in morphological and chemical groups that are spatially distributed across the pit in clusters that most likely represent individual production batches and/or the output of specific workshop units^15–17^. Given the uneven presence/absence of chromium on the weapons, we hypothesised that only certain weapon batches, perhaps those made in a particular workshop, had been treated with chromium. However, spatial analysis shows that the distribution of chromium-bearing weapons is largely random (Fig. 5) and it does not correlate with the distribution of the weapon production batches identified previously^15–17^. This evidence suggests that the chromium presence is not the result of deliberate action. Furthermore, as discussed below, there is no correlation between chromium presence and a given weapon’s state of preservation, which argues against the surface chromium deriving from deliberate CCC.

No Cr-bearing products have been employed for the conservation or storage of these artefacts, hence the possibility of chromium contamination post-excavation can be ruled out too.

We now proceed to assess other hypotheses, leading to the conclusion that the traces of chromium found on some weapons are the product of post-depositional contamination from Cr-rich lacquer.

### Chromium in the metal ores

It may be hypothesised that the chromium in some of the bronzes could derive from an impurity in the ores from which the metals making up the bronze were smelted. According to this hypothesis, chromium would have been reduced to the metallic state during smelting, and it would have then migrated to the surface of the objects during oxidation as part of a corrosion process.

The main argument against this hypothesis is the highly reducing conditions required to reduce chromium from its ore in the first instance^18,19^. The standard free energy of formation of chromium oxide is much lower than that of any oxide of copper, tin, lead or iron, i.e. the metals known to have been smelted in Qin China. As such, the reduction of chromium oxide to metal would most likely have been beyond the technical reach of Qin metallurgists. If a chromium compound was present in the ore, under typical operating conditions this would have parted into the slag phase during smelting^20^. Thus we cannot expect chromium to have been present in the metal used to make the weapons. This is consistent with numerous bulk chemical analyses of Chinese bronzes from the period, which never detect chromium in the metal^21,22^.

### Chromium in the burial soil

Researchers have hypothesised that the chromium in the bronzes could derive from the soil in which the weapons were buried^8,9^. According to this proposal, chromium oxide would have been deposited on the surface of some weapons via post-depositional contamination.

Chemical analyses of soil samples from Pits 1 and 2 show consistently low values of chromium (<100 ppm), thus making it unlikely that the enrichment on the bronzes could be explained by soil contamination (Table S3). Furthermore, the only common stable cationic form of chromium, Cr^3+^, is not very mobile in soils^23^ and therefore chromium migration from soil to metals is highly improbable. To further falsify this hypothesis, we conducted accelerated ageing experiments of bronze coupons buried in Pit 1 soil spiked with chromite (FeCr_2_O_4_), the most common Cr-bearing mineral available in nature, which occurs in northwest China. Our results show that no chromium migrated from the soil onto the bronzes, even under extreme conditions of temperature and humidity (see Supplementary Information). Overall, the hypothesis of the weapons’ surface chromium deriving from soil contamination can be put to rest.

### Chromium in the pigments

The terracotta warriors and other elements of the mausoleum are known to have been once covered by colourful natural and artificial pigments. Following Han Rubin^5^ we considered the possibility that chromium on the bronze surfaces could derive from the early use of a Cr-bearing pigment. However, no Cr-bearing pigments have been identified anywhere in the Mausoleum. This is in spite of extensive science-based research focused on polychromy, which has recorded and characterised numerous natural and artificial pigments including various compounds of lead, copper, iron, in addition to bone white, cinnabar and barium copper silicates^24–27^. Thus it is very unlikely that any pigments could be the source of chromium.

### Chromium in the lacquer

The terracotta warriors and other elements of the mausoleum were coated with one or more layers of lacquer before being painted with pigments^28–31^. Numerous additives are known historically to have been added to lacquer during manufacture, in order to facilitate the curing process or to modify its final appearance^32–34^. We hypothesised that a chromium compound could have been added to the lacquer by Qin artisans, rendering it Cr-rich and thus a potential source of chromium contamination for the weapons.

Our chemical analyses of lacquer samples show a substantial Cr content, in agreement with previous results (Table S4)^24:351,25:373^. Spectral peaks for Cr in pXRF analyses are notably higher in lacquer than in any other sample from the mausoleum analysed (Fig. 2B). Chromium is also enriched on the outer surface of the warriors, which would have been in direct contact with this Cr-rich lacquer (Table S5; Fig. 2C). This finding confirms that the lacquer constitutes the main source of chromium at the site, and adds support to our hypothesis.

We propose that not only the warriors, but also some organic materials formerly present at the site but not preserved would have been covered by Cr-rich lacquer. These lacquered elements would have included the shafts of long weapons such as lances and halberds, crossbow stocks, and sword grips and scabbards – all of which are likely to have been made of wood or bamboo^1:258–60^. The presence of chromium in these weapon parts would explain the consistent patterns of chromium presence/absence on bronze surfaces, depending on their proximity to lacquer, i.e. highly probable in sword grip fittings, shaft ferrules and those trigger parts bolted to the stock, and highly improbable in blades or arrowheads. The application of lacquer to weapon shafts and sword scabbards is documented in historical bamboo slips as well as in rare finds of weapons with their organic components preserved^35:97,^^35–37^, which renders our proposal highly plausible. This would also explain the pattern noticed in scabbard fitting 3801 mentioned above, with its inner surface in contact with the lacquered wood and hence Cr-rich, and the outer surface exposed to the soil and devoid of chromium contamination.

The source and reason for the presence of chromium in the lacquer requires further investigation. Although several Cr-compounds have been used deliberately as pigments since the 18^th^ century AD, the relatively small amounts of chromium detected in the Qin lacquer are unlikely to have conferred it any strong colour. Microscopic examination and Raman analyses of lacquer samples failed to identify any crystalline form of chromium, thus making it unlikely that a ground mineral such as chromite, crocoite (PbCrO_4_), or other water-insoluble oxide would have been mixed in. Although some plants are known to be tolerant to toxic elements such as chromium^38^ the lacquer tree is not among them^39^. Even if the ashes of a chromium hyperaccummulator plant were to have been mixed with the lacquer, a substantial quantity of these would have been needed to raise the bulk chromium in the lacquer to detectable levels. Hence, it is unlikely that chromium would have been naturally abundant in any organic ingredient. As a further hypothesis, lacquer may have been mixed with chrome alum (KCr(SO_4_)_2_), or water-soluble chromate salts that may have occurred naturally as weathering products of chromite. Chrome alum is used traditionally as a tanning agent in the treatment of leather^40^, and it is possible that Qin artisans used a similar reagent to treat their lacquer^24:351^. The presence of metal ions in lacquer significantly accelerates its curing and polymerisation, and perhaps Qin artisans intuitively noticed this, in the same way as it is exploited by modern craftsmen^41^.

## Explanations for Bronze Preservation

It might be argued that, even if surface chromium is post-depositional rather than deliberate, it could have contributed to the preservation of the weapons. Such supposition is not tenable after our analyses, however, given that there is no clear correlation between chromium presence and metal preservation, and that some of the best-preserved objects such as sword and lance blades are notoriously Cr-poor. It is therefore necessary to find alternative explanations.

Tin content is likely to have played an important role in aiding metal preservation. Several studies have noted that bronzes with higher tin levels tend to develop passive Sn-rich surface layers that prevent further corrosion^12–14^, and this phenomenon has been raised to explain the preservation of some Terracotta Army weapons, particularly those that would have been quenched^8,9^. In support of this observation, our surface chemical analyses of batches of bronze arrows showed an inverse correlation between average tin levels and their coefficients of variation, indicating that objects with higher tin had more stable compositions in the burial environment^17^. Additionally, Tylecote observed that small levels of arsenic in bronze, as found in these objects, contribute to inhibit corrosion^42^.

In addition, the nature of the soil where the bronzes were buried may have contributed to their preservation. This factor has not been considered in previous work. The Terracotta Army is located in the southern edge of the Chinese Loess Plateau, a 640,000 km^2^ area covered by silt-sized aeolian sediments that make the bulk of the soil. Large-scale models predict pH values around 8–9 for the Lintong area^43^, and this was confirmed by our on-site measurements of soil samples from Pits 1 and 2, showing pH values between 8.1 and 8.5 (Table S3). Burial soil pH is a paramount parameter predicting metal preservation, as it is correlated with redox potential, drainage conditions, biological activity and aeration. Additional characteristics of loess of potential relevance here are its low organic content and predominantly very small particle size^44,45^. We propose that the moderate basicity and low organic content of the loess would have prevented the formation of acids that would attack metal integrity. In addition, the very small particle size of the soil would have obstructed the aeration and humidity necessary for metal corrosion. Our proposal is consistent with studies in conservation science which have addressed the optimum conditions for metal preservation in burial environments, noting pH levels of 8–8.5 and small particle size as optimum^42,46,47^.

**Figure 6:**
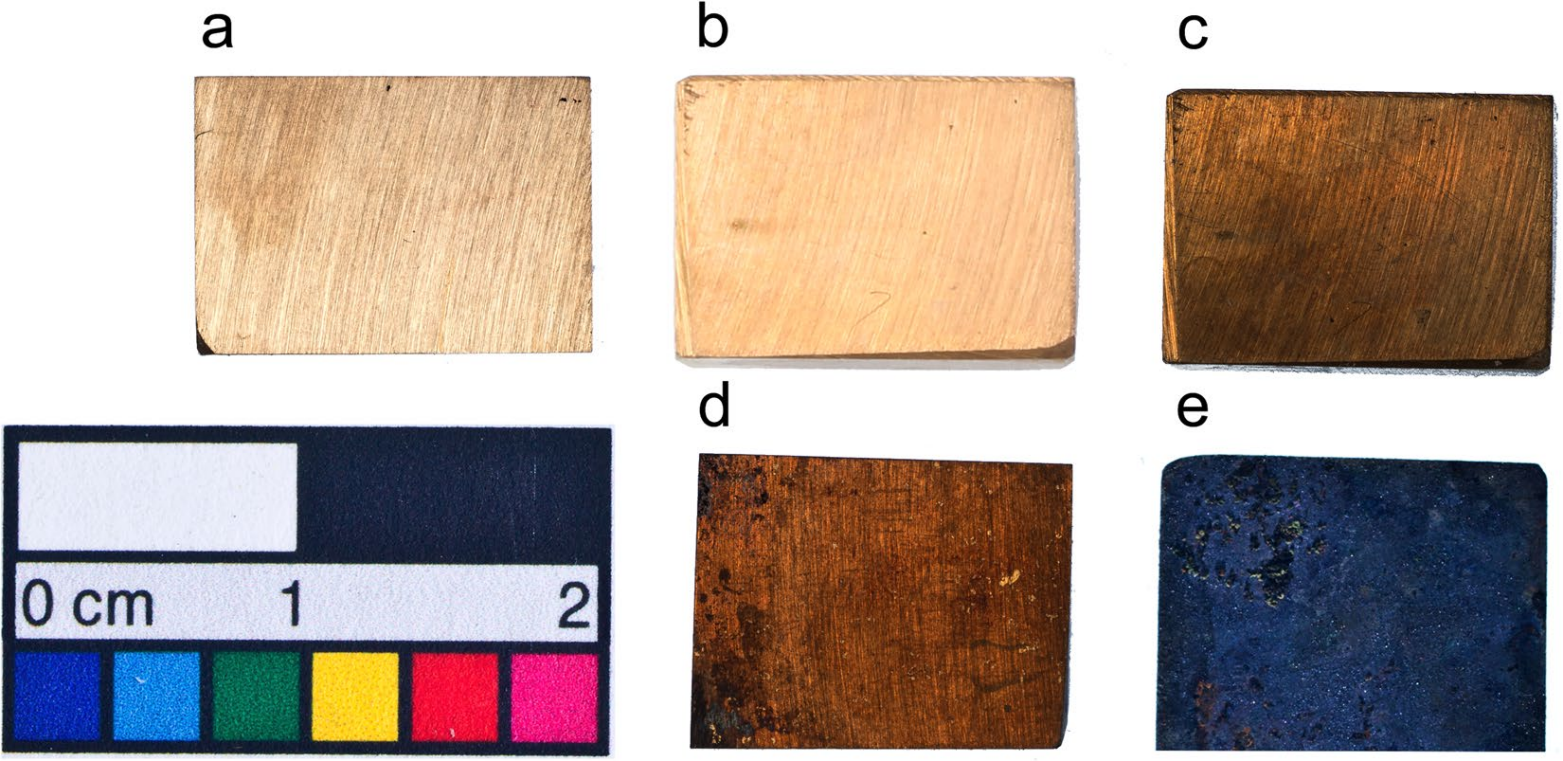
Main results of the CCC and accelerated ageing experiments on bronze tokens: (**a**) untreated bronze; (**b**) treated by CCC; (**c**) treated by CCC and aged in Terracotta Army soil; (**d**) untreated by CCC and aged in Teracotta Army soil; (**e**) untreated by CCC and aged in organic-rich soil. Note the lack of corrosion for both bronzes aged in Terracotta Army soil (see Fig. S2 for more details).

In order to verify this claim, we subjected experimental bronze tokens to accelerated ageing in an environmental chamber at 90% RH and 60 °C for four months. Both the CCC bronze tokens and the untreated bronze buried in excavation soil from Pit 1 retained equally pristine surfaces. Conversely, the control bronze token buried in an organic-rich soil with a pH of 5.9 showed visible signs of corrosion (Figs 6 and S1; Supplementary Information). The results of this experiment confirm that a key factor in the preservation of the bronzes is the nature of the soil, irrespective of the presence/absence of surface chromium.

**Figure 7:**
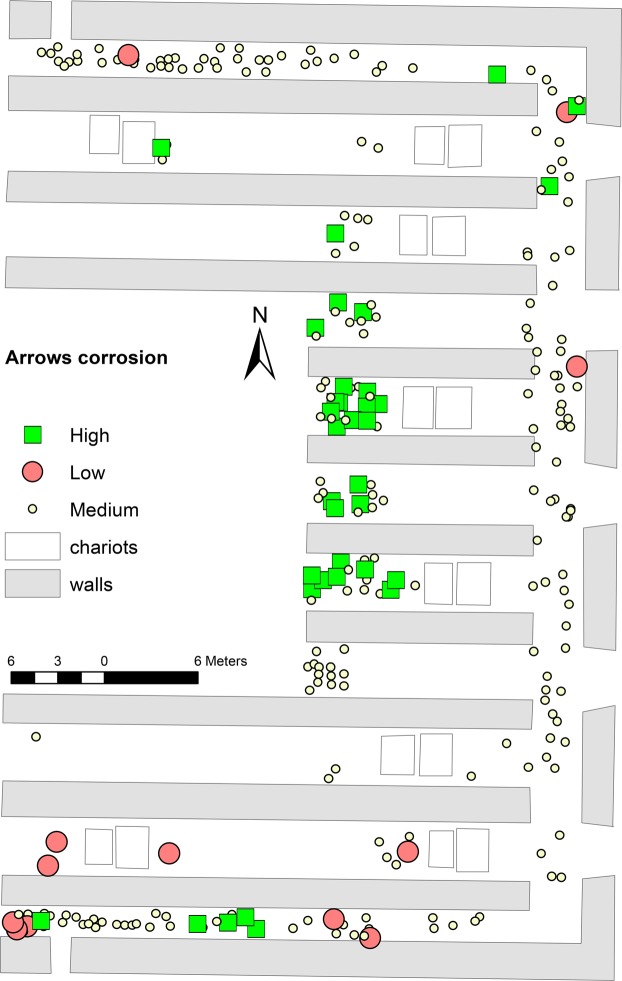
Spatial distribution of corroded vs non-corroded arrows. Distribution map of the arrow bundles excavated in the easternmost trenches of Pit 1 classified by their degree of corrosion. Photographs of the 278 arrow bundles from Pit 1 containing 90+ arrows were used for this test. For each bundle, there was one general photograph of the complete bundle and a more detailed one of a random subset of 10 arrows. Three archaeologists with experience of metals were asked to independently classify them in three groups depending on the apparent degree of corrosion: high, medium or low. When there was agreement between at least two of the observers in the classification of a given bundle as ‘low’, that bundle was labelled as ‘low corrosion’. When all three of them agreed that corrosion was ‘high’, the bundle was assigned to the ‘high corrosion’ group. The rest were labelled ‘medium corrosion’. The different thresholds were applied in order to increase the size of the ‘low corrosion’ group, as there were fewer instances of agreement among all three observers.

Finally, it is likely that taphonomic aspects to be investigated further may also have contributed to variability in metal preservation. As a first approach to explore this variable, we classified the 278 arrow bundles from Pit 1 containing 90+ arrows in three groups, according to their state of preservation. Their spatial distribution shows that the more corroded bundles cluster predominantly in the central corridors behind the chariots, whereas the best-preserved ones tend to concentrate in the southernmost corridors (Fig. 7).

## Discussion and Conclusion

Instrumental analyses of bronze weapons and weapon parts from the Terracotta Army have shown that chromium is present on the surface of some of them, but there is no association between chromium presence and state of preservation. Conversely, there is an association between chromium presence and typology, with the metal weapon parts directly associated to wooden elements such as shafts, handles or crossbow stocks showing a higher likelihood of surface chromium. Based on the high concentrations of chromium found on lacquer samples and the absence of chromium in the soil, we proposed that the chromium on some metal surfaces derives from contamination from the lacquer, and that chromium-bearing lacquer was used to coat the wooden parts of weapons.

One of the main factors responsible for the overall good preservation of metals at the site is the nature of the soil, particularly its moderately high pH, small particle size, low aeration and low organic content inhibiting corrosion processes. In addition, other parameters such as tin content and taphonomic processes are behind the variable preservation patterns detected within the site.

In conclusion, the perplexing suggestion that Qin weapon makers used an arcane chromium-based technology to prevent weapon rust has been refuted. Efforts should be made to update museum displays and other popular literature about the site with this new information. Furthermore, we predict that chromium will be detected on the surface of metal objects from other sites where they may have been in association with chromium-bearing lacquered parts, i.e. more likely on weapons than on ritual bronzes. The use of chromium-rich compounds in the manufacture of ancient lacquer should be in the agenda for future research, together with further technological study of the sharp and lustrous blades.

The First Emperor’s Mausoleum is an exceptional archaeological site representing a crucial moment in world history, and its understanding has benefitted from long-term international co-operative and interdisciplinary research. It is important that high-profile archaeological sites are studied in their wider contexts and taking advantage of comparative approaches, and that academic results are presented to the wider public. It is also critical that the aims and results of different specialist approaches are integrated. This study has provided an example of a long-standing question that could only be solved by combining studies of metal, lacquer and soil, and by placing the site in a broader context.

## Methods

### Portable X-ray flourescence (pXRF)

The majority of the pXRF analyses of metal objects were carried out using an Innov-X Systems (now Olympus) system, model Alpha, equipped with a Ag tube and a SiPIN detector with a resolution of ca. 180 eV FWHM for 5.9 kV X-rays with a beam diameter of ca. 6 mm. All analyses were conducted with the factory-built Alloys method, at 40 kV, using a 2 mm Al filter in the X-ray path for a 25 s livetime, and quantified using a fundamental parameters algorithm. A small number of analyses were carried out using a Delta Premium model from the same manufacturer, equipped with a Au tube and a silicon drift detector (SDD), and using the same analytical protocol. This instrument has a better resolution (ca. 150 eV) and a more sensitive detector, resulting in lower limits of detection (LOD). We established lower confidence LOD for both instruments at 0.1 wt%, hence surface chromium is reported as ‘present’ when detected at or above this threshold. All the analyses were conducted directly on unprepared metal surfaces, hence they cannot be assumed to accurately reflect the composition of the bulk metal.

It should be noted that the sample depth from which XRF is emitted is in the order of several tens of micrometers, and hence deeper than any potential chromium-rich layer. As such, small amounts of Cr in a very superficial layer may be diluted in the bulk analysis and fall below the pXRF LOD. This is exemplified by the analysis of sword blade 3724 where pXRF did not detect Cr but where SEM-EDS analyses, which are more superficial, did identify Cr enrichment in some areas. As such, our report of Cr presence/absence based on pXRF underestimates the real number of objects with surface Cr but the consistency of the dataset still allows us to observe clear patterns.

For ceramics, lacquer and soil, pXRF analyses were carried out using the Innov-X Systems Delta Premium model above, but using the factory-built Soils method instead, which uses a Compton-normalised algorithm for quantification. Cr was measured at 15 kV, with a 100 µm Al filter for a 40 s livetime. Instrumental performance was monitored by analysing three NIST standards (Table S1). As the set-up is specifically calibrated for minor and trace elements in silica matrices, the values for Cr obtained on lacquer samples are only reported as indicative.

### Scanning electron microscopy with energy dispersive spectrometer (SEM-EDS)

Three different instruments were employed to examine both the unprepared surfaces and epoxy-mounted polished cross-sections of samples and experimental tokens: (1) a Philips XL30 with an Oxford Instruments X-sight EDS; (2) a Hitachi S 3400 N with an Oxford Instruments X-sight EDS; and (3) a Quanta 650 with an Oxford Instruments X-Max EDS. All SEM-EDS quantitative values reported in this paper derive from the first two instruments, with quantification algorithms based on fundamental parameters and optimised with matrix-matched standards, using ZAF correction procedures built into INCA software. Typical operating parameters for EDS acquisition were: accelerating voltage of 15–20 kV, acquisition livetimes of 75–100 s, and working distance of 10 mm. A cobalt standard was analysed every 30–60 min to adjust for drift in beam intensity and optimise quantification. Analytical errors for elements in concentrations ≥1 wt% are better than 10% relative.

### Raman

Approximately 30 spots were analysed in six samples of lacquer recovered in separate locations of Pits 1 and 2, specifically looking for potential chromium compounds, but none were found. The instrument employed was a Renishaw inVia Raman Microscope with a 514 nm Nd:YAG laser, covering a range from 100 to 4500 cm^−1^.

### pH measurements of soils

A mixture of soil and deionised water in a weight ratio of 1:2 was stirred in glass beakers every 10 minutes for 1 hour, before carrying out the measurements. Three measurements were performed for each sample at intervals of 60 seconds between them. The pH meter (Labquest 2) was calibrated with two buffer solutions (pH 4.00 ± 0.01 and 10.00 ± 0.01).

## Supplementary information

Supplementary Information

## Data Availability

All data generated or analysed during this study are included in this published article and its Supplementary Information files, and/or in the bibliographic references cited where appropriate.
