# Supplementary material for: Surface chromium on Terracotta Army bronze weapons is neither an ancient anti-rust treatment nor the reason for their good preservation
Source: Sci Rep. 2019 Apr 4;9:5289. doi: 10.1038/s41598-019-40613-7 (PMC6449376; doi:10.1038/s41598-019-40613-7)
Supplement: Supplementary file 1 — Supplementary Information [file 41598_2019_40613_MOESM1_ESM.pdf]

Supplementary Information

Surface chromium on Terracotta Army bronze weapons is neither an ancient anti-rust treatment nor the reason for their good preservation

Marcos Martinón-Torres[1]*, Xiuzhen Li[2,3], Yin Xia[3], Agnese Benzonelli[2], Andrew Bevan[2], Shengtao Ma[3], Jianhua Huang[3], Liang Wang[3], Desheng Lan[3], Jiangwei Liu[3], Siran Liu[4], Zhen Zhao[3], Kun Zhao[3], Thilo Rehren[2,5]

[1]Department of Archaeology, University of Cambridge, United Kingdom.

[2]UCL Institute of Archaeology, London, United Kingdom.

[3]Emperor Qin Shihuang's Mausoleum Site Museum, Xi'an, P.R. China.

[4]Institute of Historical Metallurgy and Materials, University of Science and Technology Beijing, Beijing, P. R. China.

[5]Science and Technology in Archaeology and Culture Research Center, The Cyprus Institute, Nicosia, Cyprus

*Correspondence to: m.martinon-torres@arch.cam.ac.uk

This file includes:

Experimental design
Materials
Tables S1 to S6
Figure S1
Chromate conversion coating and accelerated ageing experiments

Experimental design

This research is part of a co-operative project concerned with the reverse engineering and production logistics of the Terracotta Army and the broader mausoleum of Qin Shihuang. For this paper, we analysed the typology, chemical composition and microstructure of bronze weapons recovered in Pit 1, to characterise their materials, technology and corrosion. We also considered the location where the artefacts were recovered within the pit during excavations. In addition, we analysed soil samples from Pits 1 and 2, and lacquer samples from terracotta warrior surfaces, to determine their chromium content and characterise soil properties that could have aided metal preservation during burial. In parallel, we carried out chromate conversion coating (CCC) experiments on bronze tokens using different protocols for comparative purposes. We conducted accelerated ageing experiments to compare the corrosion potential of the archaeological soil from Pit 1 compared to controls, and of CCC bronzes compared to controls, as well as to test the possible migration of chromium to bronze surfaces under burial conditions.

Materials

We analysed a total of 464 weapons or weapon parts by pXRF, trying to cover the spatial and typological span of the population (Table S1). Nine of these were additionally analysed by SEM-EDS, including arrowheads, ferrules, blades and a sword fitting. In addition, we analysed five samples of lacquer (Table S4) and five samples of soil (Table S3) recovered during excavations in Pits 1 and 2. We also report results of analyses of five ceramic warrior fragments (Table S5). The sampling of lacquer, soil and warrior fragments was opportunistic, based on random samples from the ongoing excavation and restoration works that could be made available to us. Additional sampling to address variability and spatial patterns in these is underway, but we believe that the results presented here suffice to support our claims.

Table S1.

Comparison between reference values for chromium and analytical results on three NIST reference materials. All values in ppm.

Standard	Reference Cr	pXRF Cr
NIST 2702	352±22	362±6
NIST 2781	202±14	195±5
NIST 2710	23±6	46±4

Table S2.

Complete list of metal artefacts analysed by pXRF, classified by typology and Museum registration number. An asterisk (*) denotes that the object or part thereof was additionally analysed by SEM-EDS. Underlined codes denote that Cr ≥0.1% was detected on the surface pXRF.

Arrowhead bundles (5-20 arrows per bundle analysed, probing head and tang separately)	Heads	1152, 1169, 1174, 1936, 1985, 1989, 1994, 2029, 2031, 2059, 2063, 2118, 2164, 2165, 2219 (3 out of 10), 2222, 2223, 2229
	Tangs	1152*, 1169, 1174, 1936, 1985, 1989, 1994, 2029*, 2031, 2059 (1 out of 10), 2063, 2118*, 2164, 2165, 2219, 2222*, 2223*, 2229
Crossbow triggers	Part A (handle)	915, 918, 926, 929, 931, 933, 954, 968, 976, 2238, 2256, 2277
	Part B (tumbler)	915, 918, 926, 929, 931, 933, 954, 968, 976, 2238, 2256, 2277
	Part C (rocking lever)	915, 918, 926, 929, 931, 933, 954, 968, 976, 2238, 2256, 2277
	Part D (bolt)	915, 918, 926, 929, 931, 933, 2256, 2277
	Part E (bolt)	915, 918, 926, 929, 931, 933, 2256, 2277
Ferrules		1677, 1678, 1686, 2383*, 2388*, 4581
Swords and lances	Blades	854, 857, 860, 997, 999, 2571, 3724*, 4099*
	Fittings	854-1, 854-2, 854-3, 857-1, 857-2, 860, 2571, 3801*

Table S3.

pH and Cr content (by pXRF) of five samples of soil from Pits 1 and 2 of the Terracotta Army site (samples 1-5), as well as organic-rich soil and Cr-spiked Terracotta Army site soil used for ageing experiments (samples 6-7). Sample 5 was not analysed for Cr.

Sample No.	pH	±	Cr (ppm)	±
Soil1 (Pit 2)	8.3	0.03	71	5
Soil2 (Pit 2)	8.1	0.01	76	5
Soil3 (Pit 1)	8.3	0.03	76	5
Soil4 (Pit 2)	8.1	0.02	65	5
Soil5 (Pit 1)	8.5	0.02		
Soil6 (organic-rich soil)	5.9	0.02	<LOD	
Soil7 (Pit 1+chromite)	8.5	0.02	1997	155

Table S4.

Cr content of the five lacquer samples analysed by pXRF.

Sample No.	Cr (ppm)	±
L1 (Pit 2)	26802	1017
L2 (Pit 2)	6120	114
L3 (Pit 1)	7582	120
L4 (Pit 2)	48410	1031
L6 (Pit 2)	916	18

Table S5.

Cr content of five warrior samples analysed, showing higher values for the outer surface (once covered with lacquer) compared to the fracture surface.

Sample No.	Location	Cr (ppm)	±
W1	outer surface	114	6
W1	fracture surface	82	6
W2	outer surface	350	8
W2	fracture surface	84	5
W3	outer surface	611	11
W3	fracture surface	92	6
W4	outer surface	575	10
W4	fracture surface	84	5
W5	outer surface	169	7
W5	fracture surface	92	6

Chromate conversion coating and accelerated ageing experiments

Pure metals were mixed to create 15 ingots of 40 g each of an alloy with composition 88Cu10Sn2Pb wt%. The composition was verified by pXRF. Each token was roughly polished and cut in 5 pieces. The resulting 75 tokens of ca. 1x1 cm were polished with 600 grit (26 µm) SiC paper. At least three replicates were created under each experimental set up.

Two different solutions were employed for chromate conversion coating (CCC) experiments:

- Solution 1: Potassium dichromate + acetic acid (s1).
  10 g of $K_2Cr_2O_7$ was mixed in 100 ml of water. On an analytical scale, acetic acid ($C_2H_4O_2$) was added to the solution until obtaining pH 2.
- Solution 2: Potassium dichromate + sulphuric acid (s2).
  10 g of $K_2Cr_2O_7$ was mixed in 100 ml of water. On an analytical scale, sulphuric acid (H_2SO_4) was added to the solution until obtaining pH 2.

The bronze tokens were treated in solutions 1 and 2 as summarised in Table S6.

Table S6.

Temperature (°C) and duration (minutes) of the CCC experiments and sample numbers for the resulting metal tokens. For example, sample B200s2 was treated in solution 2 for 200 minutes at 80°C.

	**100'**	**200'**	**500'**
**80°C**	B100	B200	
**20°C**	D100		D500

After CCC treatment, one token from each experiment was kept as reference, and two placed in a beaker buried in soil from Pit 1 of the Terracotta Army site (pH 8.5). The accelerated ageing was performed in an environmental chamber (Carbolite PF 60) with controlled relative humidity (90%) and temperature (60°C) for four months.

In addition, three control bronzes that did not undergo CCC were subjected to accelerated ageing in the same conditions, in the following media:

- the same Terracotta Army soil (pH 8.5) (Ctrl1)
- an organic-rich soil (pH 5.9) (Ctrl2)
- Terracotta Army soil spiked with ground chromite ($FeCr_2O_4$) (Ctrl3)

An assessment of the effectiveness of the different CCC treatments is beyond the scope of this paper. Overall, CCC led to slight darkening of the surfaces, particularly those performed with solution s2 and at higher temperatures. After accelerated ageing, all samples buried in Terracotta Army soil (including the control Ctrl1) appeared darker in colour but with no obvious patina or active corrosion. Conversely, the control sample buried in low pH soil (Ctrl2) appeared noticeably corroded, with a black patina and clear pitting of the surface (figure S1).

The surface of the metal sample token aged in chromite-enriched soil (Ctrl3) was subsequently analysed by pXRF and SEM-EDS. No chromium was detected on the surface.

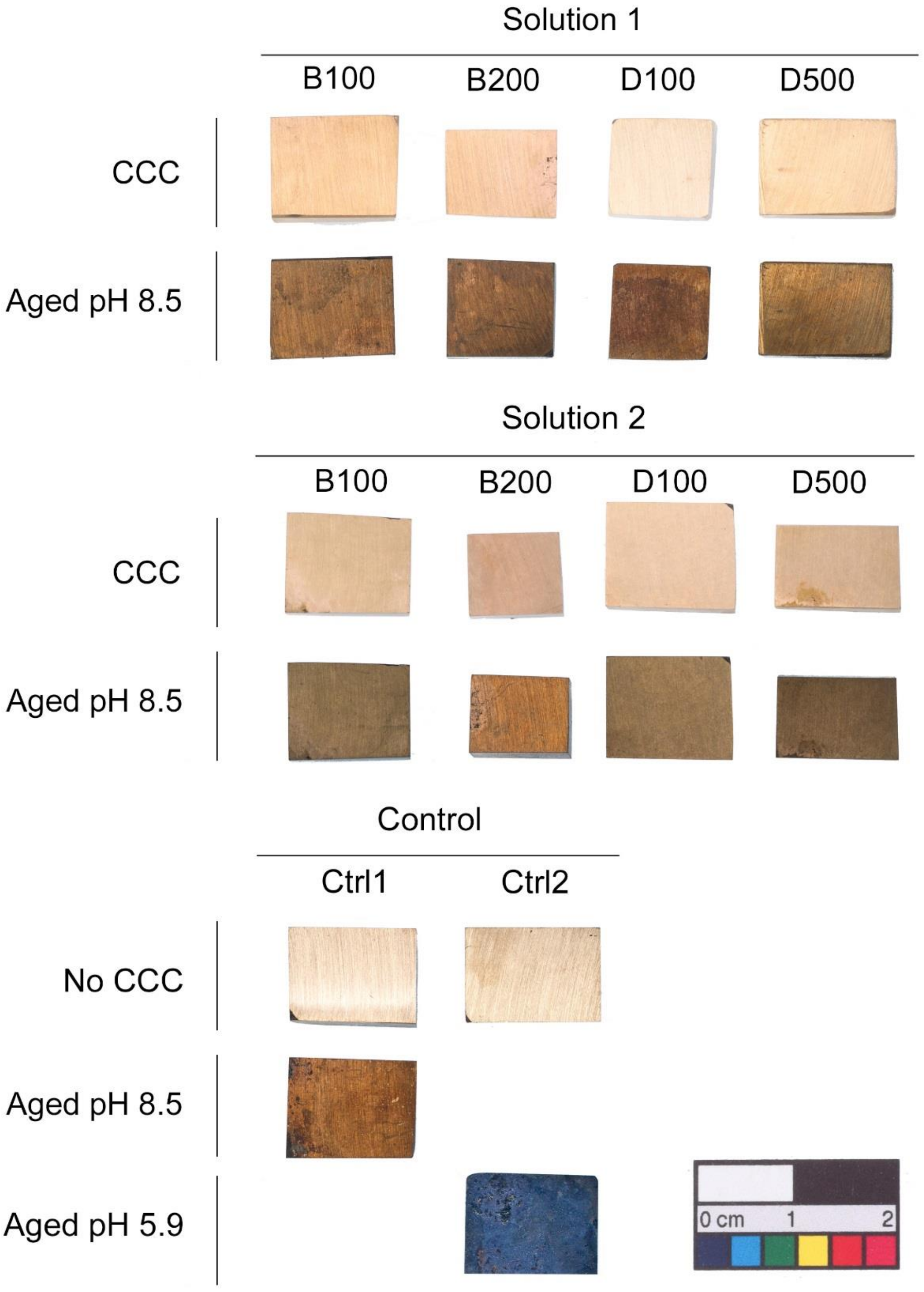

Figure S1.

Visual summary of the results of the chromate conversion coating and accelerated ageing experiments.
